# MR g-ratio-weighted connectome analysis in patients with multiple sclerosis

**DOI:** 10.1038/s41598-019-50025-2

**Published:** 2019-09-18

**Authors:** Koji Kamagata, Andrew Zalesky, Kazumasa Yokoyama, Christina Andica, Akifumi Hagiwara, Keigo Shimoji, Kanako K. Kumamaru, Mariko Y. Takemura, Yasunobu Hoshino, Kouhei Kamiya, Masaaki Hori, Christos Pantelis, Nobutaka Hattori, Shigeki Aoki

**Affiliations:** 1 Department of Radiology, Juntendo University Graduate School of Medicine, Tokyo, Japan; 2 Melbourne Neuropsychiatry Centre, Department of Psychiatry, The University of Melbourne & Melbourne Health, Parkville, VIC Australia; 3 Melbourne School of Engineering, University of Melbourne, Melbourne, Australia; 4 Department of Neurology, Juntendo University Graduate School of Medicine, Tokyo, Japan; 5 Department of Radiology, The University of Tokyo, Bunkyo, Tokyo Japan; 6 Department of Diagnostic Radiology, Tokyo Metropolitan Geriatric Hospital, Tokyo, Japan; 7 Centre for Neural Engineering, Department of Electrical and Electronic Engineering, The University of Melbourne, Carlton, VIC Australia; 8 Florey Institute of Neuroscience and Mental Health, Parkville, VIC Australia

**Keywords:** Diagnostic markers, Multiple sclerosis

## Abstract

Multiple sclerosis (MS) is a brain network disconnection syndrome. Although the brain network topology in MS has been evaluated using diffusion MRI tractography, the mechanism underlying disconnection in the disorder remains unclear. In this study, we evaluated the brain network topology in MS using connectomes with connectivity strengths based on the ratio of the inner to outer myelinated axon diameter (i.e., g-ratio), thereby providing enhanced sensitivity to demyelination compared with the conventional measures of connectivity. We mapped g-ratio-based connectomes in 14 patients with MS and compared them with those of 14 age- and sex-matched healthy controls. For comparison, probabilistic tractography was also used to map connectomes based on the number of streamlines (NOS). We found that g-ratio- and NOS-based connectomes comprised significant connectivity reductions in patients with MS, predominantly in the motor, somatosensory, visual, and limbic regions. However, only the g-ratio-based connectome enabled detection of significant increases in nodal strength in patients with MS. Finally, we found that the g-ratio-weighted nodal strength in motor, visual, and limbic regions significantly correlated with inter-individual variation in measures of disease severity. The g-ratio-based connectome can serve as a sensitive biomarker for diagnosing and monitoring disease progression.

## Introduction

Multiple sclerosis (MS) is an inflammatory demyelinating disease of the central nervous system characterized by impairments in motor, sensory, visual, and cognitive functions^1^. These symptoms are mainly caused by a disruption in the ability of nerves to conduct electrical impulses to regions with unmyelinated white matter (WM)^2^. Thus, MS can be considered a disconnection syndrome^3,4^, and brain network topology analysis can be used to provide a better understanding of the underlying mechanisms and identify important biomarkers for its diagnosis and treatment.

Recently, it has been proposed that individual brain functions are not based solely on the properties of individual regions but on the interactions within the entire network. Attempts have been made to comprehensively map the neural connections in the brain^5,6^, resulting in the formation of a connectome, which has been used to delineate network-based alterations in neurological and psychiatric disorders^7–9^. Diffusion-weighted magnetic resonance imaging (DW-MRI) can evaluate a connectome at the macroscopic level^10^. In patients with MS, connectome analysis using DW-MRI has provided new insights into disrupted structural connectivity among motor, somatosensory, visual, and limbic regions^4,11–14^. For instance, Shu *et al*. (2016, 2011) applied DW-MRI-based connectome analysis with graph theoretical analysis to investigate alterations in the network efficiency in patients with MS^4,14^. In these studies, they reported significantly decreased global and local network efficiencies in patients with MS compared with those in controls, with the most pronounced changes observed in the sensorimotor, visual, and default-mode regions, including the limbic regions^4^. They also reported reduced structural connectivity within the sensorimotor and visual regions of patients with MS compared with that within the sensorimotor and visual regions of controls^4,14^. In addition, decreased structural connectivity in limbic regions, such as hippocampus and amygdala^15^, and increased local path length, which indicate compromised network integration, have been demonstrated in patients with MS^12^. However, the precise microstructural mechanisms underlying these deficits remain unknown.

Each connection within a connectome is typically associated with a weight reflecting interregional connectivity strength. Ideally, connectivity strength should characterize biologically interpretable properties, such as axon density, myelination, and diameter, but DWI-MRI cannot provide these direct measures^16^. For example, the connectivity strength among regions are typically weighted based on the number of streamlines (NOS) intersecting a pair of regions. NOS estimated using probabilistic tractography provides information about the spatial distribution of the path with the least resistance to water diffusion. NOS has been shown to moderately correlate with connectivity strength estimated in neuronal tract tracing studies^17^, but tractography is affected by noise and orientation modeling errors^18^ as well as high rates of false positives^8,19^.

Rather than using NOS to measure connectivity strength, voxel-specific measures of diffusion anisotropy and neurite morphology can be spatially averaged across a set of streamlines associated with a specific tract to yield strength estimates with improved biological interpretability. Neurite orientation dispersion and density imaging (NODDI) is one such voxel-specific measure that provides information about the morphological structure of neurites (axons and dendrites). For each voxel, NODDI assumes a three-compartment biophysical tissue model, including intracellular fluid, extracellular fluid, and cerebrospinal fluid (CSF)^20^. Recently, for more detailed characterization of cell structure in the brain, microstructural information from NODDI was combined with myelin-sensitive MRI, such as magnetization transfer (MT) imaging, to yield voxel-specific estimates of the g-ratio, which is defined as the ratio of axon diameter (excluding myelin) to myelinated fiber diameter (including myelin)^21^.

Originally, the g-ratio of a myelinated axon was directly visualized and measured using electron microscopy^22,23^. This method obtains the diameters of axons and myelinated axons of an immunohistochemically processed specimen. The g-ratio determines the neuron conduction velocity^24,25^ and influences normal brain functions^26^. The g-ratio dynamically changes during normal development^27^ and in demyelinating diseases such as MS^28^. Therefore, it could be used as a measure of myelination, demyelination, and remyelination. However, calculating the g-ratio using electron microscopy is not only time-consuming but also is expensive and limited to *ex vivo* analysis. Thus, it cannot be used for whole-brain analysis. These issues have been the driving force for *in vivo* estimation of the g-ratio using MRI.

However, it has been very difficult to estimate the g-ratio using MRI because the optimum spatial resolution of MRI is a few millimeters, whereas axon diameter evaluation requires submicron-scale resolution. Therefore, in recent years, an approach for measuring area-weighted g-ratio in voxels has been proposed^21^. This is termed the magnetic resonance (MR) g-ratio and is calculated with the myelin volume fraction (MVF) and axon volume fraction (AVF) using myelin-sensitive and diffusion-sensitive methods, respectively. The MR g-ratio is an *in vivo* imaging technique that enables whole-brain analysis and has been shown to be sensitive to the presence of demyelinating lesions in MS^29^. Estimated g-ratio values can be averaged across all voxels that intersect the set of streamlines interconnecting a pair of regions, thereby yielding a myelin-specific measure of connectivity strength. This can be repeated for all pairs of regions to map connectomes that explicitly relate to myelin, referred to as a g-ratio-based connectome.

In this study, we mapped connectomes based on the g-ratio to clarify the role of demyelination in brain network disturbances in patients with MS. The use of connectomes based on the g-ratio was first demonstrated in healthy individuals by Mancini *et al*.^30^. To the best of our knowledge, the present study is the first to map connectomes based on the g-ratio in a clinical population. We hypothesized that the g-ratio is more sensitive to connectivity deficits than the conventional measures of connectivity strength. To test this hypothesis, we compared connectivity strength obtained using a g-ratio-based connectome with that obtained using a conventional NOS-based connectome. In particular, we mapped whole-brain connectomes and evaluated the differences in terms of interregional connectivity strength between the MS and control groups. Further, we analyzed the correlation between connectivity strength and the disease severity of MS.

## Results

### WM lesion load

**Figure 1:**
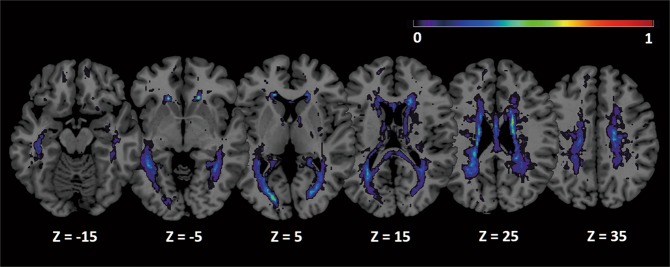
Mean WM lesion probability distribution overlaid on the Colin brain^82^ in MNI space. The color overlay created on the MNI standard brain represents the probability of lesion occurrence (frequency) at each voxel location. Warm colors indicate lesion loci common to multiple individuals. Images are shown in radiological convention. *Abbreviations: MNI, Montreal Neurological Institute; WM, white matter*.

Cerebral lesions were present in all patients with MS. The WM lesion probability map shown in Fig. 1 quantifies the proportion of patients with lesions encompassing each WM voxel. While WM lesions were predominantly located in periventricular deep WM, substantial inter-individual variation was evident in terms of lesion extent and location. The mean total volume of WM lesions in the MS group was 4.3 ± 4.8 mL.

### Disrupted WM connections

**Figure 2:**
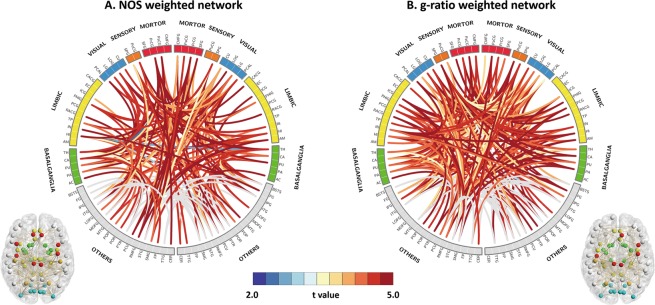
Comparison of subnetworks with significantly altered connectivity in each network. (**A**) Subnetworks with significantly decreased NOS-weighted connectivity in patients with MS versus controls. (**B**) Subnetworks with significantly increased g-ratio-weighted connectivity in patients with MS versus controls (See correspondence of abbreviations with anatomical regions in Supplementary Table S4). *Abbreviations: NOS, number of streamlines; MS, multiple sclerosis*.

For NOS- and g-ratio-based connectomes, the null hypothesis of equality in connectivity strength between the MS and control groups was rejected (*P* < 0.05). For the g-ratio-based connectome, tract-averaged g-ratio values increased in the MS group compared with those in the control group (*P* < 0.05) for connections associated with motor, somatosensory, visual, and limbic regions (Fig. 2A, Supplementary Table S1). In particular, the network-based statistic (NBS) identified a subnetwork comprising 244 connections among 73 regions associated with increased g-ratio values in the MS group (*P* = 0.04). These included 8 motor, 4 somatosensory, 8 visual, and 16 limbic regions. For the NOS-based connectome, NBS also identified a comparable subnetwork comprising 189 connections among 70 regions with reduced NOS in the MS group compared with that in the control group (*P* = 0.04; Fig. 2B, Supplementary Table S1). These included 6 motor, 3 somatosensory, 8 visual, and 13 limbic regions. Connectivity strength was averaged across all connections within each subnetwork, yielding a subnetwork-averaged NOS and g-ratio value for each individual. Inter-individual variation in terms of these subnetwork-averaged NOS and g-ratio values significantly correlated with WM lesion volumes (NOS: *P* = 0.007, r = −0.69; g-ratio: *P* = 0.006, r = −0.70) but not with disease duration or expanded disability status scale (EDSS) score.

### Nodal strength

**Figure 3:**
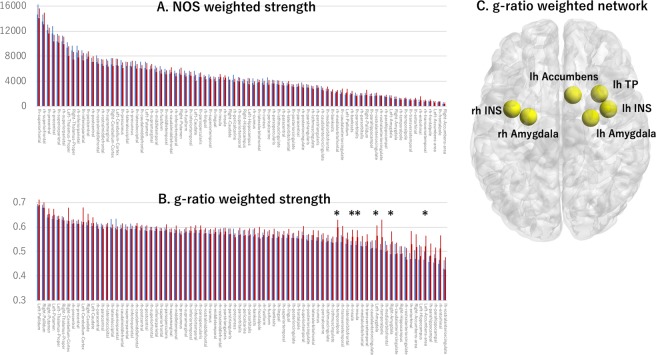
Graphical representation of the comparison between NOS-weighted and g-ratio-weighted strength distribution. NOS-weighted strength distribution (**A**) and g-ratio weighted strength distribution (**B**) in patients with MS (blue bars) and controls (red bars). (**C**) This is a representative image showing regions with increased g-ratios in patients with MS versus controls (*P* < 0.05, FDR corrected). *Value differed significantly (P < 0.05, FDR corrected) between patients with MS and healthy controls. Abbreviations: FDR, false detection rate; NOS, number of streamlines; MS, multiple sclerosis.

NOS-weighted nodal strength tended to reduce in the MS group relative to the control group in brain regions such as the left inferior parietal lobe, left medial orbitofrontal lobe, left insular, left thalamus, right thalamus, right caudate, right precentral lobe, and right insular (uncorrected *P* < 0.05; Fig. 3A, Supplementary Table S2). However, no significant differences remained after false discovery rate (FDR) correction. In contrast, for the g-ratio-based connectome, several regions were found to have significantly increased nodal strength in the MS group relative to the control group (Fig. 3B, Table 1). The most prominent increases in nodal strength in the MS group were evident in the limbic regions, including the bilateral insular, bilateral amygdala, left temporal pole, and left accumbens (Fig. 3C, Table 1).

**Table 1:** Regions with significant between-group differences in terms of g-ratio-based nodal strength.

Regions	Controls	Patients with MS	t-value	*P*-value	Cohen’s *d*
Left temporal pole	0.54 (0.04)	0.60 (0.03)	−4.19	0.00029*	−1.64
Left accumbens	0.47 (0.03)	0.52 (0.04)	−3.58	0.00138*	−1.40
Right insula	0.53 (0.02)	0.56 (0.03)	−3.43	0.00204*	−1.34
Right amygdala	0.49 (0.04)	0.54 (0.04)	−3.32	0.00268*	−1.30
Left insula	0.53 (0.02)	0.56 (0.03)	−3.23	0.00333*	−1.27
Right amygdala	0.51 (0.04)	0.56 (0.04)	−3.22	0.00343*	−1.26

Notes: Patients had relapsing–remitting multiple sclerosis. Data are expressed as mean (standard deviation).

*Denotes statistical significance.

**Figure 4:**
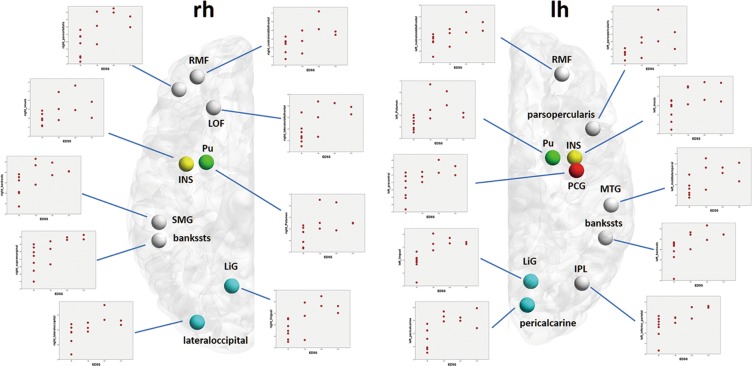
Regions where the g-ratio-weighted nodal strength was significantly correlated with the EDSS score in patients with MS. *Abbreviations: Cd, caudate; EDSS, Expanded Disability Status Scale; lh, left hemisphere; LOF, lateral orbitofrontal; MS, multiple sclerosis; PoC, postcentral gyrus; PT, pars triangularis; Pu, putamen; rh, right hemisphere; SM, supramarginal gyrus; TT, transverse temporal gyrus*.

In addition, significant positive correlations were observed between the EDSS scores and nodal strength computed using the g-ratio-based connectome in the MS group, predominantly in motor, visual, and limbic regions (Fig. 4, Table 2). For the NOS-based connectome, nodal strength did not correlate with EDSS score or disease duration.

**Table 2:** Regions where the g-ratio-based nodal strength significantly correlated with the EDSS score.

Regions	FDR-corrected *P*-value	*R*
Left lingual	0.034	0.813*
Left inferior parietal	0.034	0.783*
Left pericalcarine	0.034	0.754*
Right supramarginal	0.034	0.754*
Left bankssts	0.034	0.750*
Left rostral middle frontal	0.036	0.735*
Right lateral occipital	0.036	0.731*
Right pars orbitalis	0.036	0.721*
Left pars opercularis	0.036	0.707*
Right rostral middle frontal	0.036	0.707*
Left insula	0.036	0.698*
Left middle temporal	0.036	0.695*
Right lingual	0.036	0.695*
Left putamen	0.036	0.691*
Right putamen	0.039	0.681*
Left precentral	0.047	0.669*
Right lateral orbitofrontal	0.047	0.662*
Right bankssts	0.047	0.660*
Right insula	0.049	0.653*

Notes: *Denotes statistical significance. Abbreviations: EDSS, Expanded Disability Status Scale; FDR, false discovery rate.

## Discussion

In this study, we evaluated whether the tract-averaged g-ratio can provide a measure of connectivity strength, which is more sensitive to the effects of demyelination than the conventional measures of connectivity strength based on NOS, for connectome mapping studies. The tract-averaged g-ratio increased in the MS group relative to the control group along the motor, somatosensory, visual, and limbic subnetworks. By contrast, NOS decreased in the MS group relative to the control group in mostly the same subnetworks. Significant correlations were detected between WM lesion volumes and mean NOS or g-ratio across the subnetworks associated with significant between-group differences. Further, the g-ratio-based connectome showed significantly increased nodal strength in patients with MS, whereas no such differences were evident for the NOS-based connectome. Significant positive correlations were observed in patients with MS in terms of EDSS scores and g-ratio-weighted nodal strength in motor, visual, and limbic regions. Overall, our results indicate that the g-ratio-based connectome can provide greater sensitivity to the effects of myelin pathology on connectivity than connectomes mapped using conventional measures such as NOS.

Significant increases in the g-ratio within networks including motor, somatosensory, visual, and limbic regions in patients with MS could be responsible for the effects of WM demyelination. It is well-established that the g-ratio is a sensitive marker for myelination and demyelination^21,28,29,31^. In particular, an increased g-ratio has been shown to indicate demyelinating lesions in pathological studies using electron microscopy^28^ and MRI^21,32^. The significant negative correlation between the tract-averaged g-ratio and WM lesion volumes (i.e., number of demyelinating lesions) in the current study supports our hypothesis, with MS known to cause local demyelinating lesions^21,32^.

The NOS-based connectomes showed alterations in MS that were largely consistent with the results of previous connectomic analyses undertaken in MS^4,12,14,15^. For instance, Shu *et al*. (2011) reported decreased structural connectivity in the motor, somatosensory, and visual regions in patients with MS. They also proposed that connectome analysis is useful for the diagnosis of MS and assessment of disease progression^4^. Further, morphological and functional MRI studies have shown decreased GM volume, cortical thickness, and neuronal activity in motor, somatosensory, visual, basal ganglia, and limbic regions in patients with MS^33–37^. Previous diffusion tensor imaging studies have indicated that the alterations in WM connections are associated with these regions^38–40^. These findings suggest that structural and functional changes exist in motor, somatosensory, visual, basal ganglia, and limbic regions in patients with MS, supporting our findings. However, the connectome based on DW-MRI only provides indirect evidence of WM connections, and although DW-MRI tractography can provide an estimate of the trajectories of fiber bundles, it cannot provide direct pathological measures of axon density, diameter, and degree of myelination^18^. Therefore, the g-ratio-based connectome offers a more straightforward method for evaluating demyelinating diseases.

The g-ratio-based connectome detected significantly increased nodal strength in the MS group, whereas the NOS-based connectome detected no differences between the two groups, suggesting that the g-ratio-based nodal strength is more sensitive at detecting abnormal network characteristics in patients with MS. Remarkably, the increased g-ratio-based nodal strength was predominantly localized to the limbic system, a region where demyelinating lesions and brain atrophy tend to occur from the early stages of MS^35,41^. Abnormalities in the WM tracts connecting structures of the limbic system have also been found in early MS with diffusion tensor imaging^42^. It was also notable that the g-ratio-based nodal strength of several brain regions, such as the motor, visual, and limbic regions, were significantly correlated with EDSS scores, implying that these regions play a key role in the development of clinical symptoms in patients with MS. EDSS is a method of quantifying disability in MS by assessing impairment in motor, sensory, visual, and other functional systems^43^. Therefore, our result suggests that the g-ratio-based nodal strength facilitates MS diagnosis from early pathological stages and could be used to monitor disease progression and treatment effects in MS.

NOS-based nodal strength reportedly reduces in MS and NOS-weighted nodal strength is useful for diagnosing and monitoring disease progression in MS^13^. In the present study, NOS-weighted nodal strength in some areas tended to decrease in the MS group, but these were not significantly different than those in the control group. The conflicting findings between the present and previous studies may be related to discrepancies in patient characteristics and inherent limitations associated with the analysis of small sample sizes. For example, a previous study^13^ included patients with MS with greater disease severity (as measured by EDSS scores) and total WM lesion volume (3.5 and 10.3 mL, respectively) compared than our study (0.9 and 4.3 mL, respectively). It is likely that the patients with greater disease severity and total WM lesion volume had greater disruptions in their brain networks. Another study^44^ demonstrated a correlation between clinical symptoms and anatomical location of MS lesions, suggesting that the difference in WM lesion location also contributes to the differences between the findings of the present and previous studies. However, Shu *et al*. (2018) did not provide details on WM lesion locations^13^; therefore, we could not examine this issue.

Certain limitations of the present study necessitate following considerations. First, the sample size of this preliminary study was small. Large-scale, multicenter studies are required to confirm the usefulness of g-ratio-based connectome analysis for evaluating MS pathology. Second, only EDSS was evaluated as an indicator of disease severity. Despite being the most popular and widely used outcome measure for disease progression in patients with MS, EDSS has been reported to have notable limitations^45^. For example, it has been pointed out that some functional domains are not sufficiently evaluated, such as cognitive function, mood, and energy levels^46^. Therefore, in future research, it will be necessary to introduce a more detailed clinical evaluation that also considers the relationship of the functional domains with the pathology of brain networks. The associations between EDSS and nodal strength (Fig. 4) must be cautiously interpreted because of the limited dynamic range of the EDSS scores in this study and highly skewed distribution of scores (i.e., most individuals were associated with the lowest score). Third, graph-theoretic measures other than nodal strength, such as network efficiency and modularity, should be considered in future studies to better characterize the topological effects of MS. However, caution is required when interpreting measures based on path length in the g-ratio-based connectome because the g-ratio is not a direct estimate of conduction velocity or information transfer and may thus provide no advantage relative to NOS^30^. Ideally, both g-ratio and axon diameter are needed to measure conduction velocity^47^. Lastly, fluid-attenuated inversion recovery (FLAIR) images used for plaque detection were acquired at a slice thickness of 4 mm with a 1-mm gap because of the limitation of synthetic MRI sequence. A gap of at least 1 mm is needed to reduce the cross-talk between slices. However, the current imaging guidelines for MS recommend a slice thickness of 3 mm without a gap for 2D MR acquisition^48^. Furthermore, a previous study reported a significant (8%) increase in lesion volumes when the slice thickness of MR images was reduced from 5 to 3 mm^49^. Therefore, the lesion volume detected in our study may be underestimated. This problem should be addressed in future studies.

In conclusion, the g-ratio-based connectome can complement conventional measures of connectivity strength when assessing structural connectivity in patients with MS. Our data suggest that the g-ratio-based connectome has the potential to be used as a biomarker for disease diagnosis, particularly at early disease stages, and for monitoring disease progression.

## Methods

### Participants

We prospectively included 14 females with relapsing–remitting MS (mean age 42.8 ± 5.0 years) from May to November 2016. All patients were diagnosed by an experienced neurologist (KY) according to the 2010 modified McDonald criteria^50^. Disease severity was evaluated using EDSS^43^. The main demographic and clinical characteristics, including EDSS score and disease duration of patients, were also assessed by KY. For comparison, we recruited 14 age- and sex-matched healthy controls (mean age 43.2 ± 14.4 years) with no history of neurological disease, psychiatric disease, drug abuse, head trauma, or seizures and no contraindications for MRI. The clinical and demographic characteristics are shown in Table 3.

**Table 3:** Participant characteristics.

	Controls	Patients	*P*-value
Number	14	14	
Age (years)	43.2 ± 14.4 (22–47)	42.8 ± 5.0 (24–44)	0.83
Sex (male/female)	0/14	0/14	1
Disease duration (years)	NA	9.6 ± 6.0 (3–22)	NA
EDSS	NA	0.9 ± 1.1 (0–3)	NA

All data are expressed as mean ± standard deviation. Minimum and maximum values for demographic and clinical values (age, disease duration and EDSS) are also provided. Abbreviations: EDSS, expanded disability status scale; NA, not applicable.

The study was approved by the Institutional Review Board of Juntendo University Hospital, Japan, and was conducted in accordance with The Code of Ethics of the World Medical Association (Declaration of Helsinki) for experiments involving humans. A written informed consent was obtained for experimentation with human subjects.

### Image acquisition

MRI data were obtained using a 3.0-T system (MAGNETOM Prisma; Siemens Healthcare, Erlangen, Germany) equipped with a 64-channel head coil. Multi-shell DW-MRI and MT saturation (MTsat) images were acquired to calculate AVF and MVF, respectively.

DW-MRI was obtained at b-values of 1000 and 2000 s/mm^2^ along 64 directions uniformly distributed on a sphere, with a simultaneous multi-slice echo-planar imaging sequence in the anteroposterior phase-encoding direction. The same diffusion directions were used for all shells. Each DW-MRI acquisition was complemented with a non-DW volume (b = 0 s/mm^2^). Standard and reverse phase-encoded blipped non-DW-MRI was also obtained to correct echo-planar imaging distortions^51^. The acquisition parameters of DW-MRI were as follows: repetition time, 3300 ms; echo time, 70 ms; voxel size, 1.8 × 1.8 × 1.8 mm^3^; number of slices, 65; simultaneous multi-slice factor, 2; number of excitations, 1; and acquisition time, 6.25 min. The multi-shell DW-MRI data were visually checked in all three orthogonal views to confirm that there were no severe artifacts due to missing signals, gross geometric distortion, or bulk motion. Then, the data were corrected for eddy currents, susceptibility-induced geometric distortions, and inter-volume motion using the EDDY and TOPUP toolboxes^52^.

The dual excitation three-dimensional (3D) multi-echo fast low-angle shot sequences were performed with predominant T1, proton density (PD), and MT weighting for calculating the MTsat index. MTsat imaging was developed to improve the MT ratio (MTR) by separating MTR from the longitudinal relaxation rate (R_1_)^53^. MTsat shows higher contrast in the brain than MTR^53^ and has been shown to correlate well with quantitative MT measures^54^. It has also been shown that MTsat is less sensitive to variations among MR imaging systems than the MTR parameter and was more effective in differentiating patients with MS from healthy controls^55^. The excitation of MT-weighted images was preceded by an off-resonance Gaussian-shaped RF pulse under the following conditions: frequency offset from water resonance, 1.2 kHz; pulse duration, 9.984 ms; and nominal flip angle, 500°. Other acquisition parameters for MTsat were as follows: MT-off and MT-on scanning [repetition time (TR)/echo time (TE) = 24/2.53 ms, flip angle = 5°] and T1-weighted imaging (TR/TE = 10/2.53 ms, flip angle = 13°); parallel imaging using GRAPPA factor 2 in the phase-encoding direction; 7/8 partial Fourier acquisition in the partition direction; bandwidth, 260 Hz/pixel; slice thickness, 1.8 mm; number of slices, 104; field of view (FOV), 224 × 224 mm; matrix, 128 × 128; and total acquisition time, 6 min 25 s.

To enable the estimation of cortical parcels and tissue segmentation (FreeSurfer), T1-weighted images (T1WI) were also acquired using 3D magnetization-prepared rapid gradient-echo sequence. The acquisition parameters were as follows: repetition time, 15 ms; echo time, 3.54 ms; inversion time, 1100 ms; voxel size, 0.86 × 0.86 × 0.86 mm^3^; and acquisition time, 5.14 min.

To acquire lesion segmentation maps, axial QRAPMASTER (quantification of relaxation times and PD by multi-echo acquisition of saturation recovery using turbo spin-echo readout) pulse sequence was performed for all patients. Details of the QRAPMASTER sequence and post-processing methods are described previously^56,57^. The parameters used for QRAPMASTER were as follows: TR, 4250 ms; TE, 22/99 ms; delay time, 170/620/1970/4220 ms; FOV, 230 × 186 mm; matrix, 320 × 260; echo train length, 10; bandwidth, 150 Hz/pixel; parallel imaging acceleration factor, 2; slice thickness/gap, 4.0/1.0 mm; number of sections, 30; and acquisition time, 5 min 8 s. Synthetic T1-weighted and FLAIR images were produced from calculated R1, R2, and PD by virtually setting TR/TE to 500/10 ms and 1500/75 ms with an inversion time of 3000 ms using SyMRI version 8.0 (SyntheticMR, Linköping, Sweden)^58^.

#### MVF calculation

First, the MTsat data were analyzed to calculate MVF using an in-house MATLAB script based on a previously described theory^53^. First, for each voxel, the apparent longitudinal relaxation rate ${R}_{1app}$ was calculated as follows:

1 $${R}_{1{\rm{app}}}=\frac{1}{2}\frac{{S}_{{\rm{T}}1}{{\rm{\alpha }}}_{{\rm{T}}1}/{{\rm{TR}}}_{{\rm{T}}1}-{S}_{{\rm{PD}}}{\alpha }_{{\rm{PD}}}/{{\rm{TR}}}_{{\rm{PD}}}}{{S}_{{\rm{PD}}}/{\alpha }_{{\rm{PD}}}-{S}_{{\rm{T}}1}/{\alpha }_{{\rm{T}}1}}$$

where ${S}_{{\rm{T}}1}$ and ${S}_{{\rm{PD}}}$ denote the signal intensities of T1- and PD-weighted images, respectively; ${{\rm{TR}}}_{{\rm{T}}1}$ and ${{\rm{TR}}}_{{\rm{PD}}}$ denote the TR of T1- and PD-weighted images, respectively; and ${\alpha }_{{\rm{T}}1}$ and ${\alpha }_{{\rm{PD}}}$ denote the excitation flip angles of T1- and PD-weighted images, respectively.

Second, apparent signal amplitude ${A}_{app}$ was calculated as follows:

2 $${A}_{app}={S}_{{\rm{PD}}}{S}_{{\rm{T}}1}\frac{{{\rm{TR}}}_{{\rm{PD}}}{\alpha }_{{\rm{T}}1}/{\alpha }_{{\rm{PD}}}-{{\rm{TR}}}_{{\rm{T}}1}{\alpha }_{{\rm{PD}}}/{\alpha }_{{\rm{T}}1}}{{S}_{{\rm{T}}1}{{\rm{TR}}}_{{\rm{PD}}}{\alpha }_{{\rm{T}}1}-{S}_{{\rm{PD}}}{{\rm{TR}}}_{{\rm{T}}1}{\alpha }_{{\rm{PD}}}}$$

Third, apparent MTsat ${\delta }_{app}$ was calculated as follows:

3 $${\delta }_{app}=({A}_{app}{\alpha }_{{\rm{MT}}}/{S}_{{\rm{MT}}}-1){R}_{1app}{{\rm{TR}}}_{{\rm{MT}}}-{{\alpha }_{{\rm{MT}}}}^{2}/2$$

where ${S}_{{\rm{MT}}}$, ${{\rm{TR}}}_{{\rm{MT}}}$, and ${\alpha }_{{\rm{MT}}}$ denote signal intensity, TR, and excitation flip angle of the MT-weighted image, respectively.

MTsat is superior to conventional MTR imaging in terms of relaxation rate, inhomogeneity of RF transmit, and receive field^53,59^. Further, to further improve spatial uniformity, we corrected small residual higher-order dependencies of the MTsat on the local RF transmit field, as suggested by Weiskpof *et al*.^60^, as follows:

4 $$M{T}_{sat}=\frac{{\delta }_{app}(1-0.4)}{1-0.4R{F}_{local}}$$

where RF_local_ is the relative local flip angle α compared with the nominal flip angle. Dual-angle method was used to calculate RFl_ocal_^61^. Additionally, we added two B1 maps using echo-planar imaging with flip angles of 10° and 20° acquired in approximately 10 s each. The first image was obtained after excitation with a flip angle α and second after excitation with a flip angle 2α. The first and second images had a magnitude proportional to sin(α) and sin(2α), respectively. The ratio of the two acquisitions was subsequently formed as follows:

5 $$\frac{\sin \,\alpha }{\sin \,2\alpha }\,=\,\frac{1}{2\,\cos \,\alpha }$$

from which the local flip angle α was calculated.

The MTsat used in this study required a calibration factor to estimate MVF. We determined the calibration factor based on all healthy controls involved in this study as a linearly proportional relationship between MVF and ${\delta }_{app}$, an absolute quantitative marker of myelin^54^. The calibration factor of 0.1 was subsequently used to obtain a g-ratio of 0.7 in the corpus callosum as previously suggested^62,63^. Therefore, to estimate MVF maps, we multiplied all voxels in the ${\delta }_{app}$ map by 0.1.

#### AVF calculation

AVF was estimated based on parameters of the NODDI model^20^. In particular, the intracellular volume fraction (Vic) and isotropic water diffusion volume fraction (Viso), indicating free water, were calculated using AMICO^64^, enabling AVF estimation as follows^21^:

6 $$AVF\,=\,(1-MVF)(1-Viso)Vic$$

AVF was independently estimated for each voxel. These calculations were performed using custom MATLAB functions (MathWorks, Natick, Massachusetts). The images were linearly registered to a common space using FLIRT^65^ before calculation.

#### MRI g-ratio calculation

A g-ratio was calculated using MVF and AVF for each voxel, generating an *in vivo* whole-brain g-ratio map using the following equation^21^:

7 $${\rm{g}}-{\rm{ratio}}=\sqrt{AVF/(MVF+AVF)}$$

Before obtaining g-ratio maps, MVF maps were first affine-aligned to the corresponding non-gradient-weighted MRI images using a statistical parametric mapping software (SPM12, Wellcome Department of Cognitive Neurology, UK; http://www.fil.ion.ucl.ac.uk/spm) This was independently repeated for each individual, and spatial correspondence between voxels was ensured on the AVF and MVF maps.

### Measurements of WM lesion volume and distribution

Given that the presence of demyelinating lesions can affect connectome mapping, we evaluated the distribution and volume of WM lesions. For all patients with MS, we automatically segmented WM lesions on synthetic FLAIR images using a lesion prediction algorithm^66^, as implemented in the Lesion Segmentation Toolbox, Version 2.0.15 (http://www.applied-statistics.de/lst.html)^67^. This algorithm comprises a binary classifier in the form of a logistic regression model trained on the data of 53 patients with MS with severe lesion patterns. As covariates for this model, we used a similar lesion belief map with the lesion growth algorithm^67^ and spatial covariate that considered voxel-specific changes in lesion probability. The parameters of this model fit were used to segment lesions by providing an estimate for the lesion probability per voxel. All lesion maps were visually inspected by a neuroradiologist (AH). The total brain WM lesion volume was calculated by multiplying the lesion area by slice thickness.

Synthetic T1-weighted images of each patient were spatially normalized to Montreal Neurological Institute space, and deformation fields were saved, which were then applied to lesion maps. Because synthetic images derived from QRAPMASTER are inherently aligned^56^, no registration was required between synthetic T1-weighted images and lesion maps created on synthetic FLAIR. Normalized lesion maps were summated for all patients to create aggregate lesion maps.

### Pre-processing for connectome

**Figure 5:**
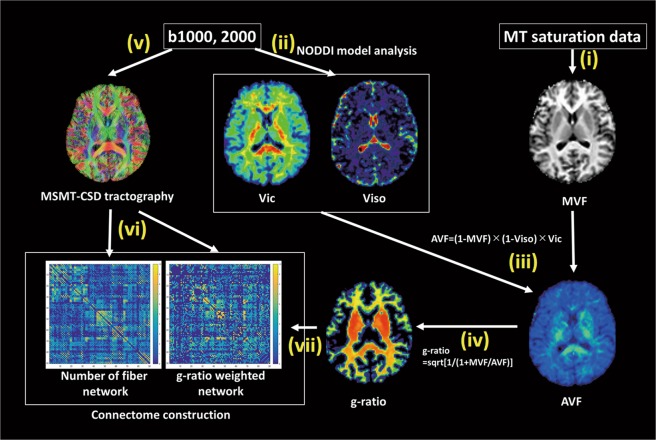
Schematic overview of the pre-processing pipeline for obtaining NOS-weighted and g-ratio-weighted connectivity matrices. NOS-weighted and g-ratio-weighted network were mapped according to the following steps. (i) MVF maps were generated from MTsat data (T1-weighted, PD-weighted, and MT-weighted images); (ii) NODDI-derived metrics, Vic and Viso, were calculated from the multi-shell DW-MRI data; (iii) AVF maps were estimated from Vic, Viso, and MVF data; (iv) g-ratio maps were calculated from AVF and MVF maps in a voxel-by-voxel manner; (v) whole-brain probabilistic tractography was performed with MSMT-CSD using multi-shell DW-MRI data and 84 connectome nodes on the Desikan–Killiany cortical atlas; (vi) NOS between each pair of nodes was enumerated; and (vii) g-ratio values were averaged across all voxels intersecting the set of streamlines interconnecting a pair of regions to yield a myelin-specific connectivity weight to contrast with NOS. This was repeated for all pairs of regions. *Abbreviations: AVF, axon volume fraction; DW, diffusion-weighted; MVF, myelin volume fraction; MSMT-CSD, multi-shell multi-tissue constrained spherical deconvolution; MT, magnetization transfer; NODDI, neurite orientation dispersion and density imaging; NOS, number of streamlines; PD, proton density; Vic, intra cellular volume fraction; Viso, isotropic water diffusion volume fraction*.

Figure 5 shows a schematic overview of the processing pipeline for obtaining NOS- and g-ratio-weighted connectivity matrices. DW-MRI data were preprocessed using the Functional MRI of the Brain (FMRIB) Software Library version 5.0.9^68^. First, for each subject, we used the nonlinear boundary-based registration approach in FMRIB to align 3D-T1WIs to the corresponding b0 map. Second, the Brain Extraction Tool^69^ was used to remove non-brain tissue from 3D-T1WIs. Third, the tissue probability maps of WM, cortical gray matter (GM), deep GM, and CSF were obtained using the FMRIB Automated Segmentation Tool^70^. Lastly, tissue probabilities of deep GM for all voxels within the brain were obtained using the FMRIB Integrated Registration and Segmentation Tool^71^. These tissue probability maps were processed for the multi-shell, multi-tissue constrained spherical deconvolution (MSMT-CSD) and anatomically constrained tractography framework.

### Node definition

The default FreeSurfer reconstruction pipeline^72^ was used to delineate 84 regions based on the Desikan–Killiany cortical atlas segmentation (Supplementary Table S5)^73^. Because of the high variability in spatial location and extent of subcortical GM parcellation mapped with FreeSurfer^72^, the subcortical GM data provided by FreeSurfer parcellation were replaced by the subcortical GM partial volume maps obtained from the FMRIB Integrated Registration and Segmentation Tool.

### Edge definition

For each subject, the MSMT-CSD probabilistic tracking method^74^ with multi-shell DW-MRI data (b = 0, 1000, and 2000 s/mm^2^) was used to generate whole-brain tractograms. Anatomically constrained tractography^75^ and spherical deconvolution informed filtering of tractograms^76^ were applied to reduce bias in the streamline density of longer fiber pathways^75^. While streamline filtering methods can alleviate biases, it is important to note that they can also compromise the specificity with which connectome pathology can be localized^19^. The MRtrix 3.0 software package (Brain Research Institute, Melbourne, Australia, http://www.brain.org.au/software/) was used to generate tractograms.

For MSMT-CSD tracking, multiple response functions were estimated as a function of the b-value and tissue type. First, voxels were assigned to WM if the fractional anisotropy was >0.7 and tissue probability was >0.95 for WM (see section 2.7). Second, the DW-MRI profile was reoriented to ensure that the principal axis of diffusion was aligned. Third, the WM anisotropic response functions were calculated per shell by averaging the reoriented DW-MRI profiles over these voxels. Fourth, if a tissue segmentation reported the respective tissue probability to be >95% with a fractional anisotropy of <0.2, these voxels were assigned to GM and CSF. The isotropic response functions for GM and CSF were acquired by averaging DW-MRI profiles per shell. Finally, the WM fiber orientation distribution function (fODF), GM fODF, and CSF fODF were obtained using the *dwi2fod* command with the *msmt-csd* option implemented in the MRtrix software. The maximal spherical harmonic orders (lmax) of WM, GM, and CSF were set to 8, 0, and 0, respectively.

Whole-brain MSMT-CSD tracking was performed on the WM fODFs using the second-order Integration over Fiber Orientation Distributions algorithm^77^, with the following parameters: step size, 0.9 mm; angle thresholds, 45° per step; length, 3.6–180 mm; and fiber orientation distribution threshold, 0.05. In total, 5 × 10^7^ streamlines were generated through seeding from the WM fODFs. Seeding points were determined dynamically using the SIFT model^76^. For tractogram reconstructions comprising 5 × 10^7^ streamlines, SIFT was also applied to filter the reconstruction from 5 × 10^7^ to 5 × 10^6^ streamlines.

### Connectome construction

To map the NOS-based connectome for each individual, the total number of streamlines interconnecting each pair of regions was enumerated and stored in a connectivity matrix. The g-ratio-based connectome was determined by averaging g-ratio values across all voxels intersecting the set of streamlines interconnecting a pair of regions. While averaging was performed across voxels, it is also feasible to determine a streamline-averaged g-ratio value for each streamline and subsequently average across the set of streamlines^30^. This was repeated for all pairs of regions, and the resulting tract-averaged g-ratios were stored in a connectivity matrix. Therefore, two connectivity matrices of dimension 84 × 84 were mapped for each individual, with connectivity strength estimated using the NOS- or tract-averaged g-ratio. Diagonal elements were excluded from the connectivity matrices. A NOS density threshold (T) was applied to exclude spurious links^78^. The links above the T% according to NOS were left unaltered, whereas links below the T% were set to 0. To avoid bias from adopting a single threshold, NOS-weighted nodal strengths were examined across several thresholds (10% < T < 30% in 5% steps)^79^.

### Nodal strength

For the NOS-based connectome, NOS-weighted nodal strength was calculated as ${\rm{s}}(i)={\sum }_{{\rm{j}}=1}^{{\rm{N}}}{{\rm{A}}}_{{\rm{ij}}}$, where i is a given node, A_ij_ is the NOS between nodes *i* and *j*, and *N* is the number of nodes in the network. Nodal strength is the simplest measure of centrality of a given node and reflects the degree of interconnectivity with other regions^80^. To estimate effect sizes for differences in nodal strength between the MS and control groups, Cohen’s *d* was computed for the mean NOS-weighted nodal strength across a range of thresholds (10% < T < 30% in 5% steps). Cohen’s *d* was the largest for a threshold of 30% (Supplementary Table S3); therefore, this was applied as the threshold for the NOS-weighted nodal strength. Nodal strengths were computed using relevant functions in the MATLAB Brain Connectivity Toolbox (http://www.brain-connectivity-toolbox.net/).

For the g-ratio-based connectome, nodal strength was calculated as ${\rm{g}}(i)=\frac{{\sum }_{j=1}^{N}{A}_{ij}{G}_{ij}}{{\sum }_{j=1}^{N}{A}_{ij}}$, where *i* is a given node and *G*_*ij*_ is the average g-ratio sampled along streamlines between the nodes *i* and *j*. Thus, nodal strength was computed as the weighted average of tract-averaged g-ratio values across all regions where the weighting factor was NOS. The NOS weighting factor accentuates the contribution of tract-averaged g-ratio values associated with connections comprising many streamlines. Connections comprising relatively few streamlines may be weak or spurious; thus, this weighting moderates the contribution of these connections.

### Identification of disrupted WM connectivity

The NBS^81^ was used to test for differences in connectivity strength between the MS and control groups. The NBS was separately performed for the NOS- and g-ratio-based connectomes to test the null hypothesis of equality in connectivity strength between the two groups for each of the 3,321 unique pairs of regions. Further details about the NBS are described elsewhere^81^. In brief, two-sample *t*-test was independently performed for each connection to test the null hypothesis of equality in connectivity strength between the MS and control groups. A primary component-forming threshold (*P = *0.05, *t = *2.06, two-tailed *t*-test) was then applied to define a set of suprathreshold edges (results across different thresholds are reported in Supplementary Table S4). Next, the number of links (or size) of the remaining connected components in the network was stored. The statistical significance of the size of each connected component was evaluated with respect to an empirical estimate of the null distribution of maximal component sizes (5000 permutations), with the component size measured as the number of edges it comprised. Any connected component was reported if it remained significant at a *P*-value of < 0.05 after family-wise error correction.

### Statistical analysis

Statistical analyses of demographic and clinical variables were performed using IBM SPSS for Windows (version 23.0; IBM Corp., Armonk, NY, USA). Demographic and clinical variables, except the EDSS score, were confirmed to be normally distributed by Kolmogorov–Smirnov test. Between-group differences were analyzed using Student’s *t*-tests for continuous variables (e.g., age, WM lesion load, and graph-theoretic metrics) and chi-square tests for sex. Because the EDSS score was not normally distributed, Spearman’s rank correlation coefficient was used to test for relationships between brain measures (e.g., WM lesion load and nodal strength) exhibiting significant between-group differences and any clinical measures (e.g., disease duration and EDSS score). FDR was used to correct for multiple comparisons using a significance threshold of *P* < 0.05.

## Supplementary information

Supportive_informations

## Data Availability

The datasets generated during and/or analyzed during the current study are available from the corresponding author on reasonable request.

## References

[1] Noseworthy JH, Lucchinetti C, Rodriguez M, Weinshenker BG (2000). Multiple sclerosis. N. Engl. J. Med..

[2] Filippi M (2012). Association between pathological and MRI findings in multiple sclerosis. Lancet Neurol..

[3] Passamonti L (2009). Neurobiological mechanisms underlying emotional processing in relapsing-remitting multiple sclerosis. Brain.

[4] Shu N (2011). Diffusion tensor tractography reveals disrupted topological efficiency in white matter structural networks in multiple sclerosis. Cereb. Cortex.

[5] Filippi M (2013). Assessment of system dysfunction in the brain through MRI-based connectomics. Lancet Neurol..

[6] Sporns O, Tononi G, Kotter R (2005). The human connectome: A structural description of the human brain. PLoS Comput. Biol..

[7] Fornito A, Zalesky A, Breakspear M (2015). The connectomics of brain disorders. Nat. Rev. Neurosci..

[8] Zalesky A (2016). Connectome sensitivity or specificity: which is more important?. NeuroImage.

[9] Fornito A, Zalesky A, Pantelis C, Bullmore ET (2012). Schizophrenia, neuroimaging and connectomics. NeuroImage.

[10] Craddock RC (2013). Imaging human connectomes at the macroscale. Nat. Methods.

[11] Li Y (2013). Diffusion tensor imaging based network analysis detects alterations of neuroconnectivity in patients with clinically early relapsing-remitting multiple sclerosis. Hum. Brain Mapp..

[12] Nigro S (2015). Structural ‘connectomic’ alterations in the limbic system of multiple sclerosis patients with major depression. Mult. Scler..

[13] Shu N (2018). Progressive brain rich-club network disruption from clinically isolated syndrome towards multiple sclerosis. NeuroImage Clin..

[14] Shu N (2016). Disrupted topological organization of structural and functional brain connectomes in clinically isolated syndrome and multiple sclerosis. Sci. Rep..

[15] Zhou F (2015). Disconnection of the hippocampus and amygdala associated with lesion load in relapsing-remitting multiple sclerosis: a structural and functional connectivity study. Neuropsychiatr. Dis. Treat..

[16] Jones DK, Knosche TR, Turner R (2013). White matter integrity, fiber count, and other fallacies: the do’s and don’ts of diffusion MRI. NeuroImage.

[17] Calabrese E, Badea A, Cofer G, Qi Y, Johnson GA (2015). A diffusion mri tractography connectome of the mouse brain and comparison with neuronal tracer data. Cereb. Cortex.

[18] Sotiropoulos SN, Zalesky A (2019). Building connectomes using diffusion MRI: why, how and but. NMR Biomed..

[19] Sarwar T, Ramamohanarao K, Zalesky A (2018). Mapping connectomes with diffusion MRI: deterministic or probabilistic tractography?. Magn. Reson. Med..

[20] Zhang H, Schneider T, Wheeler-Kingshott CA, Alexander DC (2012). NODDI: practical *in vivo* neurite orientation dispersion and density imaging of the human brain. NeuroImage.

[21] Stikov N (2015). *In vivo* histology of the myelin g-ratio with magnetic resonance imaging. NeuroImage.

[22] Hildebrand C, Hahn R (1978). Relation between myelin sheath thickness and axon size in spinal cord white matter of some vertebrate species. J. Neurol. Sci..

[23] Friede RL, Beuche W (1985). Combined scatter diagrams of sheath thickness and fibre calibre in human sural nerves: changes with age and neuropathy. J. Neurol. Neurosurg. Psychiatry..

[24] Rushton WA (1951). A theory of the effects of fibre size in medullated nerve. J. Physiol..

[25] Waxman SG (1980). Determinants of conduction velocity in myelinated nerve fibers. Muscle Nerve.

[26] Dean DC (2016). Mapping an index of the myelin g-ratio in infants using magnetic resonance imaging. NeuroImage.

[27] Schroder JM, Bohl J, von Bardeleben U (1988). Changes of the ratio between myelin thickness and axon diameter in human developing sural, femoral, ulnar, facial, and trochlear nerves. Acta Neuropathol..

[28] Albert M, Antel J, Bruck W, Stadelmann C (2007). Extensive cortical remyelination in patients with chronic multiple sclerosis. Brain Pathol..

[29] Hagiwara A (2017). Analysis of white matter damage in patients with multiple sclerosis via a novel *in vivo* MR method for measuring myelin, axons, and g-ratio. AJNR Am. J. Neuroradiol..

[30] Mancini M (2017). Introducing axonal myelination in connectomics: A preliminary analysis of g-ratio distribution in healthy subjects. NeuroImage.

[31] Dupree JL, Feinstein DL (2018). Influence of diet on axonal damage in the EAE mouse model of multiple sclerosis. J. Neuroimmunol..

[32] Hagiwara A (2017). Synthetic MRI in the detection of multiple sclerosis plaques. AJNR Am. J. Neuroradiol..

[33] Steenwijk MD (2016). Cortical atrophy patterns in multiple sclerosis are non-random and clinically relevant. Brain.

[34] Wen J, Yablonskiy DA, Salter A, Cross AH (2017). Limbic system damage in MS: MRI assessment and correlations with clinical testing. PloS One.

[35] Calabrese M (2015). Regional distribution and evolution of gray matter damage in different populations of multiple sclerosis patients. PloS One.

[36] Ceccarelli A (2008). A voxel-based morphometry study of grey matter loss in MS patients with different clinical phenotypes. NeuroImage.

[37] Rocca MA (2010). Default-mode network dysfunction and cognitive impairment in progressive MS. Neurology.

[38] Reich DS (2008). Corticospinal tract abnormalities are associated with weakness in multiple sclerosis. AJNR Am. J. Neuroradiol..

[39] Dasenbrock HH (2011). Diffusion tensor imaging of the optic tracts in multiple sclerosis: association with retinal thinning and visual disability. J. Neuroimaging.

[40] Yoshida M (2013). Diffusional kurtosis imaging of normal-appearing white matter in multiple sclerosis: preliminary clinical experience. Jpn. J. Radiol..

[41] Audoin B (2010). Atrophy mainly affects the limbic system and the deep grey matter at the first stage of multiple sclerosis. J. Neurol. Neurosurg. Psychiatry..

[42] Koenig KA (2015). The relationship between cognitive function and high-resolution diffusion tensor MRI of the cingulum bundle in multiple sclerosis. Mult. Scler..

[43] Kurtzke JF (1983). Rating neurologic impairment in multiple sclerosis: an expanded disability status scale (EDSS). Neurology.

[44] Altermatt A (2018). Clinical correlations of brain lesion location in multiple sclerosis: voxel-based analysis of a large clinical trial dataset. Brain Topogr..

[45] Meyer-Moock S, Feng YS, Maeurer M, Dippel FW, Kohlmann T (2014). Systematic literature review and validity evaluation of the Expanded Disability Status Scale (EDSS) and the Multiple Sclerosis Functional Composite (MSFC) in patients with multiple sclerosis. BMC Neurol..

[46] van Munster CE, Uitdehaag BM (2017). Outcome measures in clinical trials for multiple sclerosis. CNS Drugs.

[47] Paus T, Pesaresi M, French L (2014). White matter as a transport system. Neuroscience.

[48] Wattjes MP (2015). Evidence-based guidelines: MAGNIMS consensus guidelines on the use of MRI in multiple sclerosis–establishing disease prognosis and monitoring patients. Nat. Rev. Neurol..

[49] Molyneux PD (1998). The effect of section thickness on MR lesion detection and quantification in multiple sclerosis. AJNR Am. J. Neuroradiol..

[50] Polman CH (2011). Diagnostic criteria for multiple sclerosis: 2010 revisions to the McDonald criteria. Ann. Neurol..

[51] Andersson JL, Graham MS, Zsoldos E, Sotiropoulos SN (2016). Incorporating outlier detection and replacement into a non-parametric framework for movement and distortion correction of diffusion MR images. NeuroImage.

[52] Andersson JL, Sotiropoulos SN (2016). An integrated approach to correction for off-resonance effects and subject movement in diffusion MR imaging. NeuroImage.

[53] Helms G, Dathe H, Kallenberg K, Dechent P (2008). High-resolution maps of magnetization transfer with inherent correction for RF inhomogeneity and T1 relaxation obtained from 3D FLASH MRI. Magn. Reson. Med..

[54] Campbell JSW (2018). Promise and pitfalls of g-ratio estimation with MRI. NeuroImage.

[55] Zhou LQ (2004). A new method for analyzing histograms of brain magnetization transfer ratios: comparison with existing techniques. AJNR Am. J. Neuroradiol..

[56] Hagiwara A (2017). SyMRI of the Brain: Rapid quantification of relaxation rates and proton density, with synthetic MRI, automatic brain segmentation, and myelin measurement. Invest. Radiol..

[57] Warntjes JB, Leinhard OD, West J, Lundberg P (2008). Rapid magnetic resonance quantification on the brain: Optimization for clinical usage. Magn. Reson. Med..

[58] Wallaert L (2017). The advantage of synthetic MRI for the visualization of anterior temporal pole lesions on double inversion recovery (DIR), phase-sensitive inversion recovery (PSIR), and myelin images in a patient with CADASIL. Magn. Reson. Med. Sci..

[59] Helms G, Dathe H, Dechent P (2010). Modeling the influence of TR and excitation flip angle on the magnetization transfer ratio (MTR) in human brain obtained from 3D spoiled gradient echo MRI. Magn. Reson. Med..

[60] Weiskopf N (2013). Quantitative multi-parameter mapping of R1, PD(*), MT, and R2(*) at 3T: a multi-center validation. Front. Neurosci..

[61] Morrell GR, Schabel MC (2010). An analysis of the accuracy of magnetic resonance flip angle measurement methods. Phys. Med. Biol..

[62] Mohammadi S (2015). Whole-brain *in-vivo* measurements of the axonal g-ratio in a group of 37 healthy volunteers. Front. Neurosci..

[63] Hori M (2018). Application of quantitative microstructural MR imaging with atlas-based analysis for the spinal cord in cervical spondylotic myelopathy. Sci. Rep..

[64] Daducci A (2015). Accelerated microstructure imaging via convex optimization (AMICO) from diffusion MRI data. NeuroImage.

[65] Jenkinson M, Bannister P, Brady M, Smith S (2002). Improved optimization for the robust and accurate linear registration and motion correction of brain images. NeuroImage.

[66] Egger C (2017). MRI FLAIR lesion segmentation in multiple sclerosis: Does automated segmentation hold up with manual annotation?. Neuroimage Clin..

[67] Schmidt P (2012). An automated tool for detection of FLAIR-hyperintense white-matter lesions in Multiple Sclerosis. NeuroImage.

[68] Greve DN, Fischl B (2009). Accurate and robust brain image alignment using boundary-based registration. NeuroImage.

[69] Smith SM (2002). Fast robust automated brain extraction. Hum. Brain Mapp..

[70] Zhang Y, Brady M, Smith S (2001). Segmentation of brain MR images through a hidden Markov random field model and the expectation-maximization algorithm. IEEE Trans. Med. Imaging.

[71] Patenaude B, Smith SM, Kennedy DN, Jenkinson M (2011). A Bayesian model of shape and appearance for subcortical brain segmentation. NeuroImage.

[72] Dale AM, Fischl B, Sereno MI (1999). Cortical surface-based analysis. I. Segmentation and surface reconstruction. NeuroImage.

[73] Desikan RS (2006). An automated labeling system for subdividing the human cerebral cortex on MRI scans into gyral based regions of interest. NeuroImage.

[74] Jeurissen B, Tournier JD, Dhollander T, Connelly A, Sijbers J (2014). Multi-tissue constrained spherical deconvolution for improved analysis of multi-shell diffusion MRI data. NeuroImage.

[75] Smith RE, Tournier JD, Calamante F, Connelly A (2012). Anatomically-constrained tractography: improved diffusion MRI streamlines tractography through effective use of anatomical information. NeuroImage.

[76] Smith RE, Tournier JD, Calamante F, Connelly A (2013). SIFT: Spherical-deconvolution informed filtering of tractograms. NeuroImage.

[77] Tournier, J., Calamante, F., Connelly, A. Improved probabilistic streamlines tractography by 2nd order integration over fibre orientation distributions. *Proc. 18th Annual Meeting of the Intl. Soc. Mag. Reson. Med*. (ISMRM) 1670 (2010).

[78] Rubinov M, Sporns O (2010). Complex network measures of brain connectivity: uses and interpretations. NeuroImage.

[79] Zhang J (2011). Disrupted brain connectivity networks in drug-naive, first-episode major depressive disorder. Biol. Psychiatry.

[80] Watts DJ, Strogatz SH (1998). Collective dynamics of ‘small-world’ networks. Nature.

[81] Zalesky A, Fornito A, Bullmore ET (2010). Network-based statistic: identifying differences in brain networks. NeuroImage.

[82] Van Essen DC (2001). Mapping visual cortex in monkeys and humans using surface-based atlases. Vision Res..

