# Supplementary material for: MR g-ratio-weighted connectome analysis in patients with multiple sclerosis
Source: Sci Rep. 2019 Sep 18;9:13522. doi: 10.1038/s41598-019-50025-2 (PMC6751178; doi:10.1038/s41598-019-50025-2)
Supplement: Supplementary file 1 — Supportive_informations [file 41598_2019_50025_MOESM1_ESM.docx]

**MR g-ratio-weighted connectome analysis in patients with multiple sclerosis**

Koji Kamagata, Andrew Zalesky, Kazumasa Yokoyama, Christina Andica, Akifumi Hagiwara, Keigo Shimoji, Kanako K Kumamaru, Mariko Y Takemura, Yasunobu Hoshino, Kouhei Kamiya, Masaaki Hori, Christos Pantelis, Nobutaka Hattori, and Shigeki Aoki

**Supplementary Table S1**. Networks that were significantly different between the MS and control groups identified using network-based statistical analysis.

| **Networks and connections** | **T value** |
| --- | --- |
| **Network using g-ratio weighted imaging (*p* = 0.04)** | |
| Left putamen to left accumbens | 4.31 |
| Left accumbens to right caudate | 4.07 |
| Right thalamus proper to right fusiform | 4.07 |
| Left precuneus to right hippocampus | 3.85 |
| Left middle temporal to left accumbens area | 3.83 |
| Right thalamus proper to right postcentral | 3.83 |
| Left superior parietal to left thalamus proper | 3.64 |
| Left thalamus proper to left putamen | 3.64 |
| Left pars triangularis to left thalamus proper | 3.62 |
| Left putamen to left hippocampus | 3.62 |
| Left lingual to left superior parietal | 3.59 |
| Right thalamus proper to right lingual | 3.56 |
| Right putamen to right inferior temporal | 3.51 |
| Left inferior temporal to left putamen | 3.48 |
| Left temporal pole to left hippocampus | 3.47 |
| Right thalamus proper to right hippocampus | 3.45 |
| Right medial orbitofrontal to right middle temporal | 3.44 |
| Left superior temporal to left putamen | 3.38 |
| Left pars triangularis to left putamen | 3.37 |
| Right putamen to right superior temporal | 3.35 |
| Left rostral middle frontal to right caudal middle frontal | 3.31 |
| Right putamen to right temporal pole | 3.31 |
| Right hippocampus to right lingual | 3.28 |
| Right thalamus proper to right superior parietal | 3.25 |
| Left putamen to right fusiform | 3.24 |
| Right thalamus proper to right precentral | 3.24 |
| Left middle temporal to left putamen | 3.23 |
| Right putamen to right caudal middle frontal | 3.21 |
| Right caudate to right postcentral | 3.19 |
| Right putamen to right superior parietal | 3.19 |
| Right thalamus proper to right pars triangularis | 3.14 |
| Right hippocampus to right postcentral | 3.14 |
| Right middle temporal to right supramarginal | 3.14 |
| Left temporal pole to left pallidum | 3.09 |
| Left superior parietal to left putamen | 3.07 |
| Right putamen to right postcentral | 3.07 |
| Right putamen to right pars orbitalis | 3.05 |
| Right fusiform to right inferior temporal | 3.03 |
| Right caudate to right superior parietal | 3.02 |
| Left isthmus cingulate to left superior parietal | 3.01 |
| Left lingual to left putamen | 3.01 |
| Left caudal middle frontal to right caudal middle frontal | 3 |
| Right putamen to right frontal pole | 3 |
| Left precuneus to right middle temporal | 2.98 |
| **Network using number of streamlines weighted (p = 0.04)** | |
| Left posterior cingulate to left superior frontal | 2.99 |
| Left pars opercularis to left insula | 3.12 |
| Left pars orbitalis to left thalamus proper | 3.19 |
| Left pars triangularis to left thalamus proper | 4.11 |
| Left postcentral to left thalamus proper | 3.02 |
| Left rostral middle frontal to left thalamus proper | 3.11 |
| Left superior frontal to left thalamus proper | 3.06 |
| Left superior parietal to left thalamus proper | 3.06 |
| Left lingual to left putamen | 3.28 |
| Left middle temporal to left putamen | 3.28 |
| Left pars orbitalis to left putamen | 3.03 |
| Left temporal pole to left putamen | 3.59 |
| Left thalamus proper to left putamen | 4.52 |
| Left inferior temporal to left accumbens | 3.67 |
| Left lingual to left accumbens | 3.25 |
| Left middle temporal to left accumbens | 3.42 |
| Left superior temporal to left accumbens | 3.95 |
| Left temporal pole to left accumbens | 3 |
| Left insula to left accumbens | 2.98 |
| Left putamen to left accumbens | 3.87 |
| Left frontal pole to right caudate | 3.14 |
| Left accumbens to right caudate | 4.13 |
| Right thalamus proper to right hippocampus | 3.91 |
| Left caudate to right accumbens | 3.17 |
| Right putamen to right accumbens | 3.33 |
| Left superior frontal to right caudal middle frontal | 3.13 |
| Right thalamus proper to right caudal middle frontal | 3.26 |
| Right thalamus proper to right inferior temporal | 3.19 |
| Right putamen to right inferior temporal | 3.64 |
| Right thalamus proper to right lingual | 3.71 |
| Left superior frontal to right medial orbitofrontal | 3.56 |
| Right inferior parietal to right medial orbitofrontal | 4.64 |
| Left hippocampus to right middle temporal | 2.97 |
| Right thalamus proper to right middle temporal | 3.69 |
| Right putamen to right middle temporal | 4.48 |
| Right accumbens to right middle temporal | 3.01 |
| Left postcentral to right paracentral | 3.38 |
| Right putamen to right pars orbitalis | 3.31 |
| Right thalamus proper to right pars triangularis | 3.13 |
| Right putamen to right pars triangularis | 3.05 |
| Right thalamus proper to right postcentral | 3.9 |
| Right putamen to right postcentral | 3.36 |
| Left caudal middle frontal to right posterior cingulate | 3.28 |
| Right thalamus proper to right precentral | 3.04 |
| Left posterior cingulate to right rostral middle frontal | 3.36 |
| Left caudal middle frontal to right superior frontal | 3.24 |
| Left rostral middle frontal to right superior frontal | 2.97 |
| Right thalamus proper to right superior parietal | 3.07 |
| Right putamen to right superior parietal | 3.28 |
| Right thalamus proper to right superior temporal | 4.01 |
| Right caudate to right superior temporal | 2.98 |
| Right putamen to right superior temporal | 4.17 |
| Left superior frontal to right frontal pole | 3.57 |
| Right thalamus proper to right frontal pole | 3.52 |
| Right putamen to right frontal pole | 3.14 |
| Right putamen to right temporal pole | 3.31 |
| Right putamen to right transverse temporal | 3.09 |
| Right pars triangularis to right insula | 3.07 |

A statistical threshold of *P* = 0.05 (*t* = 2.02, two-tailed *t*-test) was applied to determine the set of suprathreshold edges among connected components. Next, the statistical significance of the size of each observed component was evaluated with respect to an empirical null distribution of maximal component sizes obtained under the null hypothesis of random group membership (5000 permutations). *Abbreviations:* MS, multiple sclerosis; NOS, number of streamlines

**Supplementary Table S2.** Regions with significant between-group differences in NOS-based nodal strength.

| **Regions** | **Controls** | **Patients** | **Uncorrected**  ***P*-value** | **FDR-corrected *P*-value** | **Cohen’s *d*** |
| --- | --- | --- | --- | --- | --- |
| Right thalamus | 8887.29  (790.11) | 7490.86  (1191.66) | 0.0016 | 0.13 | 1.38 |
| Right caudate | 4227.57  (520.92) | 3430.79  (845.85) | 0.0076 | 0.27 | 1.13 |
| Left thalamus | 9404.71 (827.10) | 8041.93 (1546.46) | 0.0095 | 0.27 | 1.10 |
| Right precentral gyrus | 11585.71 (1218.41) | 10445.43 (963.99) | 0.0136 | 0.29 | 1.04 |
| Left inferior parietal gyrus | 8190.57 (756.44) | 7412.07 (1088.95) | 0.0440 | 0.46 | 0.83 |

All patients had relapsing–remitting multiple sclerosis. Notes: Data are expressed as mean (standard deviation). * denotes statistical significance. *Abbreviations:* FDR, false discovery rate; NOS, number of streamlines

**Supplementary Table S3.** Cohen’s *d* of the global metric across all sparsity thresholds between the MS and control groups.

|  | **Cohen’s *d*** | | | | |
| --- | --- | --- | --- | --- | --- |
| Thresholds | 10% | 15% | 20% | 25% | 30% |
| Mean NOS-weighted nodal strength | 0.334 | 0.385 | 0.424 | 0.451 | 0.470 |

*Abbreviations:* MS, multiple sclerosis; NOS, number of streamlines

**Supplementary Table S4.** Networks that significantly differed between the MS and control groups using network-based statistical analysis.

|  | **NOS-weighted connectome** | **g-ratio-weighted connectome** |
| --- | --- | --- |
| *P*-value = 0.05  T = 2.02 | **Network 1**  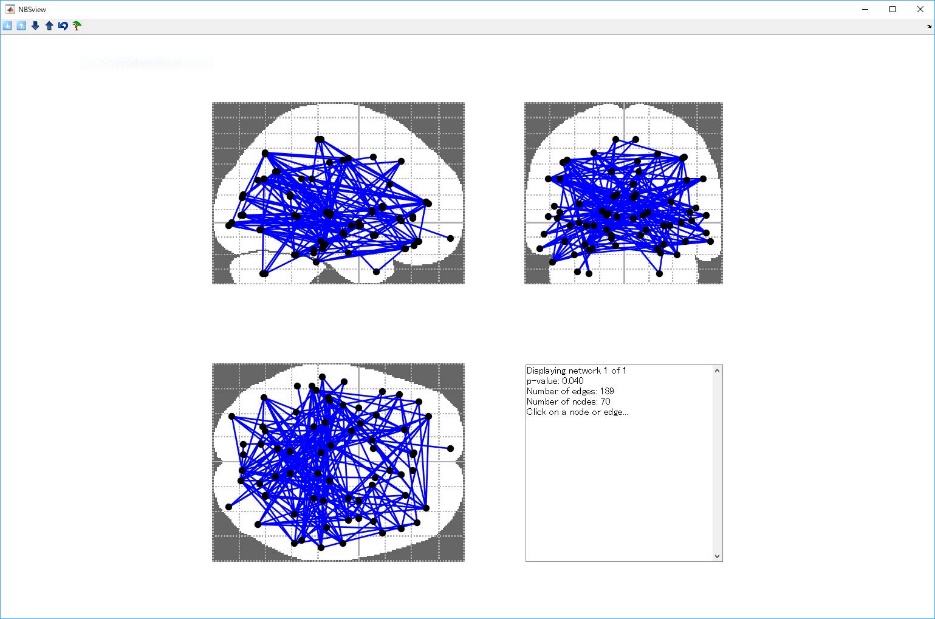 | **Network 1**  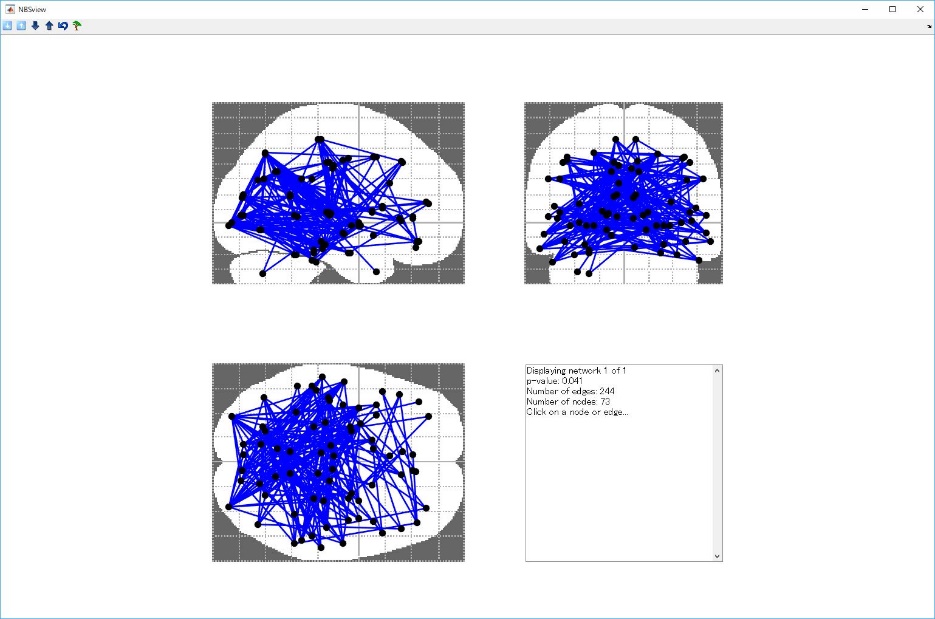 |
| *P*-value = 0.01  T = 2.78 | **Network 1**  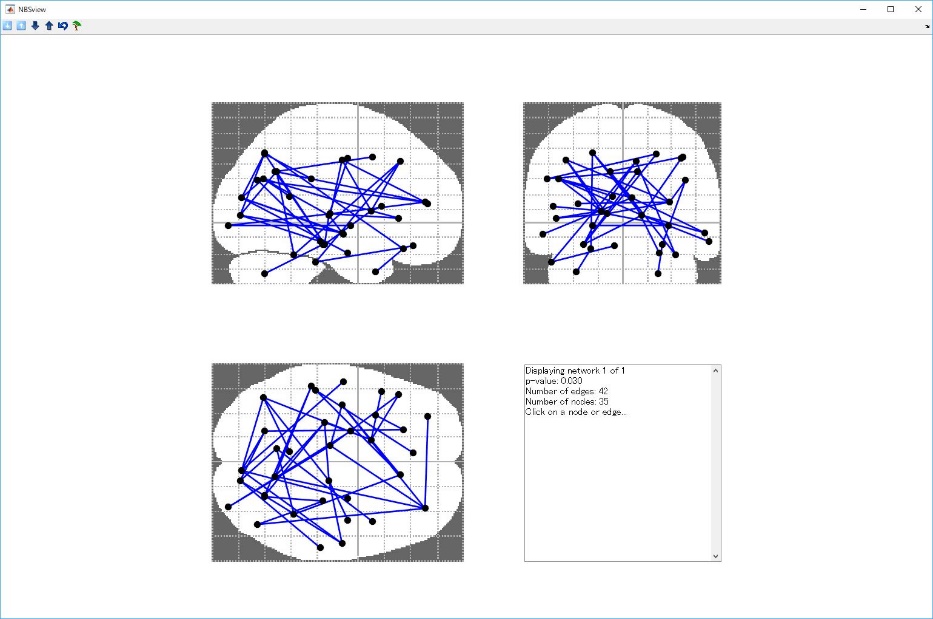 | **Network 1**  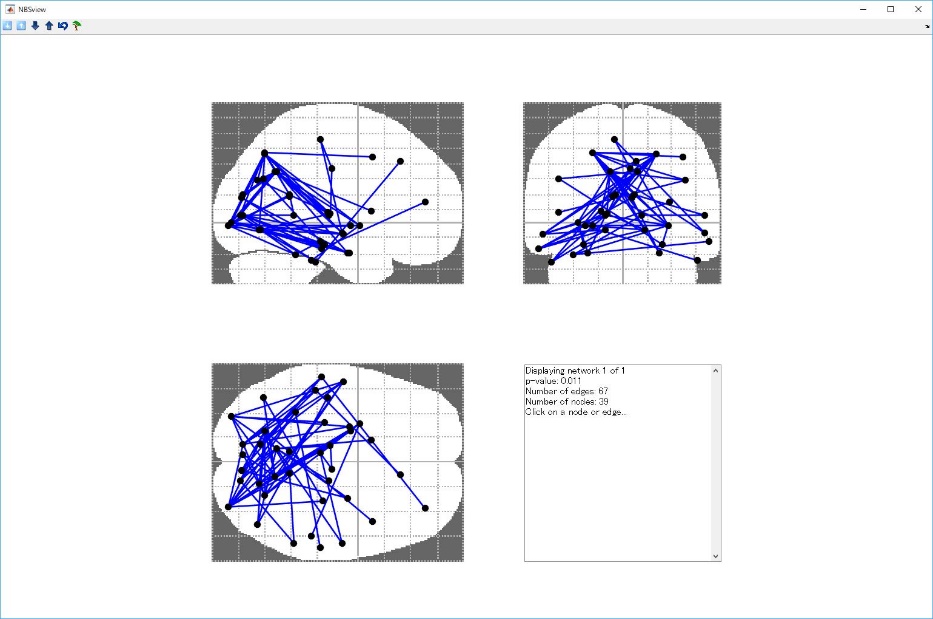 |
| *P*-value = 0.005  T = 3.07 | **Network 1**  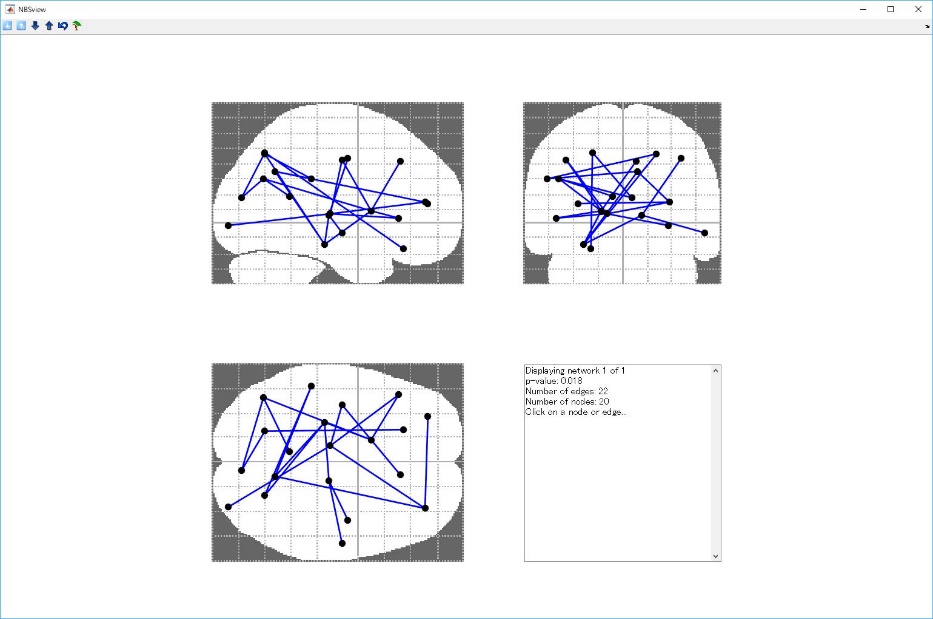 | **Network 1**  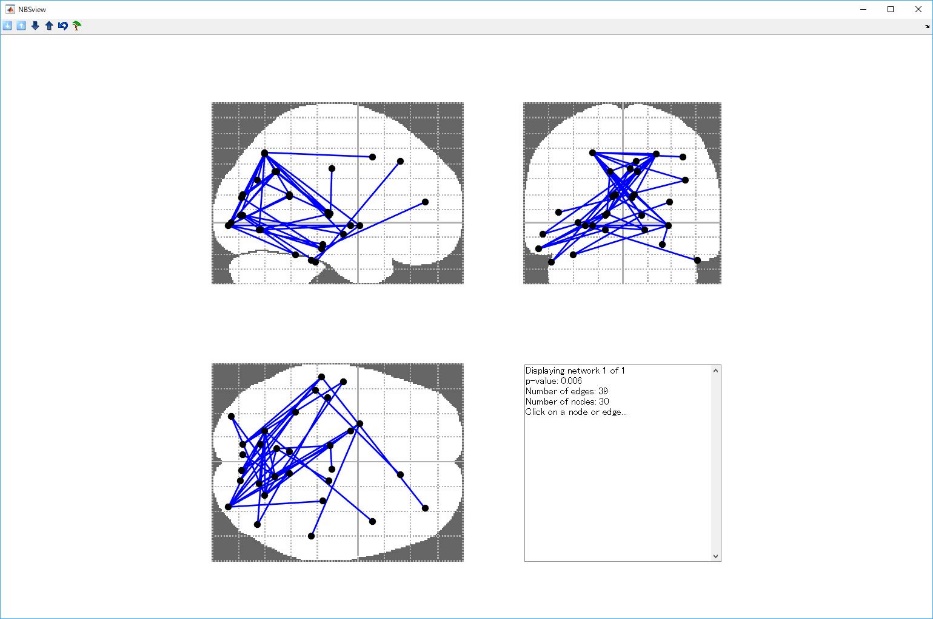 |

We explored the results of the network-based statistic using different thresholds to identify the suprathreshold edges (0.05 ≤ *P* ≤ 0.005). *Abbreviations:* MS, multiple sclerosis; NOS, number of streamlines

**Supplementary Table S5.** Anatomical cortical regions of interest used as nodes, corresponding to the Desikan–Killiany cortical atlas.

| Anatomical ROI | Label |
| --- | --- |
| Accumbens area | AC |
| Amygdala | AM |
| Banks of superior temporal sulcus | BSTS |
| Caudal anterior cingulate cortex | CACG |
| Caudal middle frontal gyrus | CMFG |
| Caudate | CA |
| Cerebellum cortex | CER |
| Cuneus cortex | SU |
| Entorhinal cortex | EC |
| Frontal pole | FP |
| Fusiform gyrus | FG |
| Hippocampus | HI |
| Inferior parietal cortex | IPG |
| Inferior temporal gyrus | ITG |
| Insula | IN |
| Isthmus cingulate cortex | ICG |
| Lateral occipital cortex | LOG |
| Lateral orbitofrontal cortex | LOFG |
| Lingual gyrus | LG |
| Medial orbitofrontal cortex | MOFG |
| Middle temporal gyrus | MTG |
| Pallidum | PA |
| Paracentral lobule | PaCG |
| Parahippocampal gyrus | PHG |
| Pars opercularis | POP |
| Pars orbitalis | POR |
| Pars triangularis | PTR |
| Pericalcarine cortex | PCAL |
| Postcentral gyrus | PoCG |
| Posterior cingulate cortex | PCG |
| Precentral gyrus | PCG |
| Precuneus cortex | PCU |
| Putamen | PU |
| Rostral anterior cingulate cortex | RACG |
| Rostral middle frontal gyrus | RMFG |
| Superior frontal gyrus | SFG |
| Superior parietal gyrus | SPG |
| Superior temporal gyrus | STG |
| Supramarginal gyrus | SMG |
| Temporal pole | TP |
| Thalamus **p**roper | TH |
| Transverse temporal cortex | TTG |
